# Transforming Care in Congenital Heart Disease: The Role of Extended Reality in Family and Trainee Education and Procedural Planning

**DOI:** 10.1007/s11886-025-02323-7

**Published:** 2025-11-27

**Authors:** Megan Gunsaulus, Julie Aldrich, Sergio A. Carrillo, John Kelly, Rehab Salah, Benjamin Blais, Arash Salavitabar

**Affiliations:** 1 The Heart Center, Nationwide Children’s Hospital, 700 Children’s Drive, Columbus, OH 43205 USA; 2 Center for Regenerative Medicine, Abigail Wexner Research Institute at Nationwide Children’s Hospital, Columbus, USA

**Keywords:** Congenital Heart Disease, Extended Reality, Virtual Reality, Patient Education, Trainee Education, Procedural Planning

## Abstract

**Purpose of Review:**

This review examines the growing role of extended reality (XR) (including virtual, augmented, and mixed reality) in the care of patients with congenital heart disease (CHD), with a focus on its use in patient education, trainee instruction, and procedural planning.

**Recent Findings:**

XR has demonstrated early success in improving patient and family understanding, enhancing trainee comprehension of cardiac anatomy, and aiding in surgical and transcatheter procedural planning for patients with complex cardiac anatomy. Studies demonstrate that XR improves visualization of 3D spatial relationships, increases confidence amongst providers and learners, and facilitates more informed preprocedural decision-making.

**Summary:**

XR addresses longstanding limitations of 2D imaging by providing interactive, patient-specific 3D environments. While most studies in this area are small and exploratory, they consistently underscore the clinical and educational value of XR in CHD care. XR is well-positioned to become a powerful tool across the continuum of CHD management.

**Supplementary Information:**

The online version contains supplementary material available at 10.1007/s11886-025-02323-7.

## Introduction

Congenital heart disease (CHD) is a uniquely complex and heterogeneous field in which understanding three-dimensional (3D) relationships is crucial for clinical decision-making and procedural planning [1]. For decades, clinicians have relied on two-dimensional (2D) imaging to understand 3D anatomy. However, translating 2D images into a 3D mental model can be cognitively demanding and error prone, potentially affecting patient care, trainee education, and communication with families [2, 3].

**Fig. 1:**
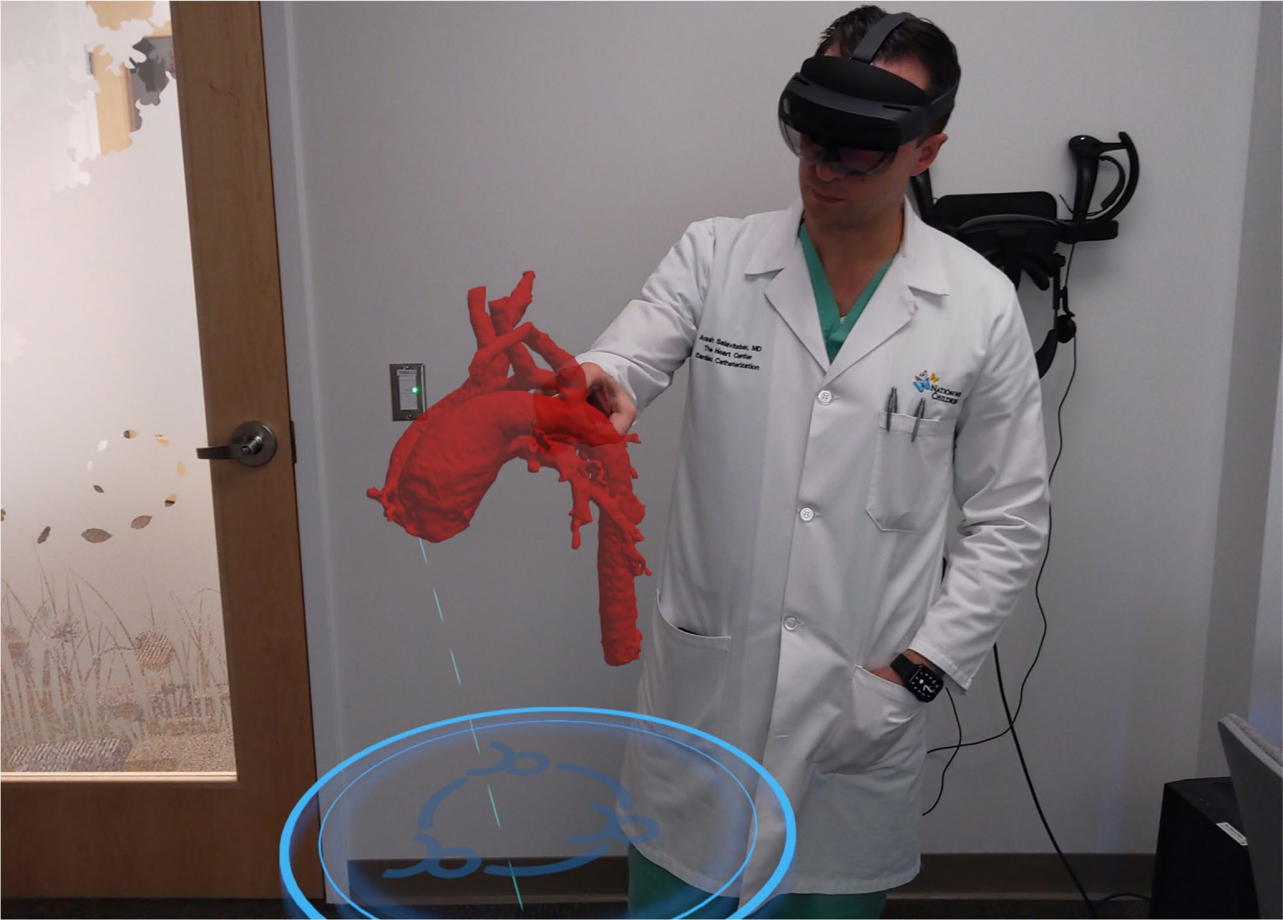
Demonstration of interactive mixed reality model using Apoqlar software (Apoqlar Medical) through a Microsoft HoloLens 2 headset (Microsoft Corporation, Washington, USA)

Extended reality (XR) refers to a spectrum of technologies that blend digital and physical environments to varying degrees and includes virtual reality (VR), augmented reality (AR), and mixed reality (MR). VR creates a fully immersive digital space for interacting with 3D models. AR overlays digital elements onto the real world, while MR goes further by anchoring virtual objects to physical structures (Fig. 1) [4, 5].

**Fig. 2:**
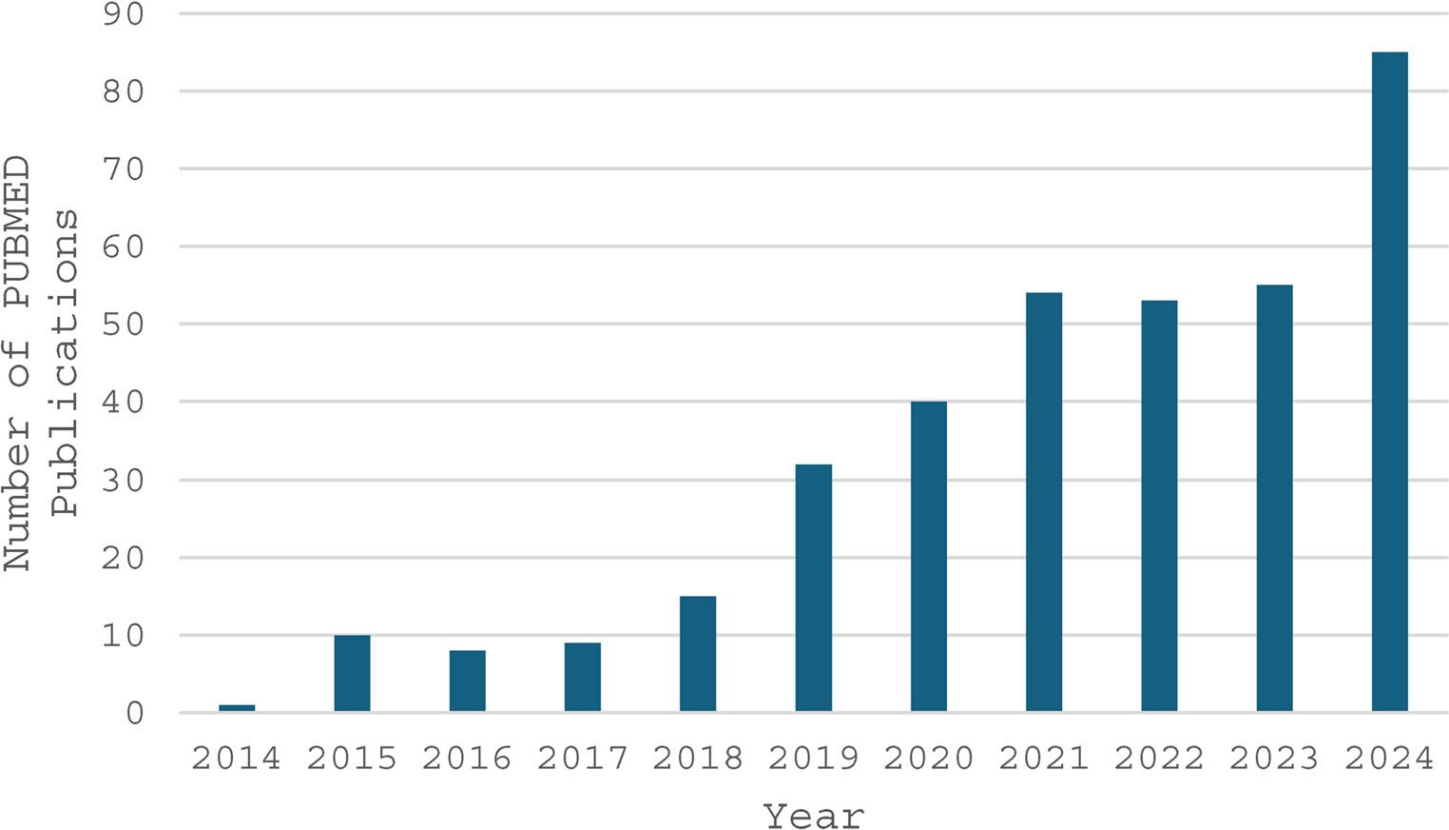
Annual trend of PubMed publications related to extended reality in the context of congenital heart disease or pediatric cardiology

3D printed cardiac models improve anatomical comprehension but require substantial time, cost, and technical expertise [6–8]. Additionally, the models are static and lack the capability for real-time manipulation [9, 10]. These limitations have led to increased interest in more interactive and cost-effective 3D visualization tools. Adoption of XR applications in CHD has grown rapidly, as demonstrated by the sharp rise in annual publications (Fig. 2). This reflects a rising recognition of XR’s potential to enhance procedural planning and education.

## Patient and Family Education

Providing effective education to patients and their families enables them to take an active, informed role in their care, which can improve outcomes and satisfaction. The most widely endorsed methods emphasize shared decision making, personalized education, and the use of diverse educational tools [11]. Traditional patient education in congenital heart disease is hindered by the complexity of cardiac anatomy and emotional or psychological responses that can limit understanding and information retention [10, 12]. The complex three-dimensional structure of the heart is often challenging to represent with 2D images, rendering conventional explanations with words and static visuals insufficient. XR offers a unique opportunity to interact with patient-specific 3D models, enhancing patient understanding and reducing the emotional distress during diagnosis and treatment [12]. High-fidelity 3D models can be constructed and viewed across a variety of XR platforms as a tool for patient education. Patients can manipulate 3D models directly, developing an intuitive understanding of the cardiac anatomy. The scale of the models can be adjusted, allowing the user to “teleport” inside the cardiac model, further exploring the anatomy and physiologic implications of the lesions [10].

Although widespread adoption and prospective studies evaluating the use of XR in CHD patient education remain limited, initial results are promising. Kieu et al. applied VR-based teaching to evaluate user experience and patient understanding of their cardiac lesion and prior surgeries. Likert scales of 1–5 (1 = strongly disagree, 5 = strongly agree) were used to score the experience of 22 patients aged 16 to 19 years. Participants reported high satisfaction with the VR-based models, with average scores exceeding 4.5 across domains, including understanding of their heart condition and surgery, overall enjoyment, and perceived value of the time involved in the experience [13]. These findings are encouraging, particularly given the growing number of adolescent and young adults living with repaired or palliated CHD. Many require lifelong follow-up, and poor health literacy may lead to interrupted care and adverse health related outcomes [14].

Cardiac diagnoses often evoke strong emotional responses, regardless of a person’s educational background or cultural context, as conditions to the heart are universally considered dangerous and life-threatening. Innately, emotions such as fear, anxiety, denial, and mistrust may arise, creating barriers to comprehension and retention. These emotional responses can limit a patient’s ability to fully participate in shared decision-making [15]. Wang et al. explored the application of VR to reduce family anxiety at the time of diagnosis of congenital heart disease. In this prospective, randomized trial, participants were divided into either a control group (provided with conventional education using paper materials) or a VR group (engaged with 3D anatomical images of the child’s heart displayed for the family using virtual reality equipment). The State-Trait Anxiety Scale, which assesses positive and negative emotions, was applied to evaluate the user experience before and after implementing the prescribed teaching. They noted a significant reduction in anxiety levels with the use of VR technology, with rates of moderate-to-severe anxiety significantly lower in the VR group (50%) compared to the control group (85%) (p-value 0.01) [16].

The clinical application of VR technologies in patient education highlights opportunities for enhanced understanding of the complex anatomy of CHD, personalized feedback through patient-specific models, improved patient satisfaction, reduced anxiety related to medical decision making, and empowerment of patients as they transition from pediatric to adult care.

## Trainee Education

The role of XR in medical education is becoming increasingly prevalent, particularly within pediatric cardiology [10, 17]. In this field, it is imperative that learners integrate understanding of complex cardiac anatomy with its associated physiology. The ability of XR to provide an interactive, immersive environment in which anatomy may be manipulated and studied in a group or individual format offers learners an enhanced understanding of intricate spatial orientations and their relationship to pathophysiology. These modalities, in turn, may improve knowledge acquisition and retention, diagnostic accuracy, skill refinement, and learner satisfaction.

Traditional methods of teaching rely on learners developing a 3D understanding of cardiac anatomy through the creation of their own mental 3D models of congenital heart defects via various imaging modalities supplemented with traditional educational material. While the understanding and integration of information from echocardiogram, angiograms, and cross-sectional imaging remains crucial, there may be significant barriers for learners. The notion of the “conceptual leap” has been described previously, referring to the difficulty learners may face when attempting to grasp certain topics presented in 2D [9, 18]. The learning process may then be unnecessarily complicated as learners attempt to conjure anatomic structures using their own spatial imagination with 2D images that do not adequately convey depth perception [3, 9]. These barriers to attaining a 3D understanding of cardiac anatomy are compounded by the steep “learning curve” in pediatric cardiology training. Depending on prior experience, learners may have limited exposure to CHD and the breadth of anatomic variety which may be encountered.

The emerging application of XR modalities within pediatric cardiology medical education is unsurprising since this technology addresses several of the previously described barriers. The use of XR is well-supported by adult learning theory [17, 19]. This pedagogy proposes that adults learn best when they understand the reason for learning a concept, engage in self-directed learning, encounter realistic or real-life patients and cases, and are motivated by internal factors [20]. The application of XR allows learners to self-dictate how they interact and explore real-world examples of complex congenital heart disease [12, 21].

The use of XR in pediatric cardiology education has been shown to be effective in teaching cardiac anatomy [21–23] and physiology [9, 17, 24]. The Stanford Virtual Heart application is one salient example [25]. With this immersive tool, learners have the ability to view and dissect various congenital heart defects and travel inside the heart itself to experience complex spatial relationships [25]. Blonsky et al. described application of the Stanford Virtual Heart model to medical students with findings of significantly improved performance on post-intervention assessments of CHD knowledge [21].

Learners exposed to XR have shown a preference for its use compared to alternative learning modalities [9, 21, 26]. For instance, Awori et al. found that resident physicians and nurse practitioners exposed to a VR model of tetralogy of Fallot reported a clear preference for and an overall greater degree of understanding with the VR model as compared to 3D printed models or traditional models of instruction [9]. d’Aiello et al. describe an innovative MR curriculum wherein medical students wore head-mounted devices and interacted directly with holographic anatomical models of sinus venosus defect with partial anomalous pulmonary venous return [26]. While the ability to describe the anatomy was comparable across all groups, trainees with the immersive MR experience were better able to answer questions regarding percutaneous treatment and potential complications and their user experience surveys were highly favorable [26].

The application of XR to procedural practice and skill acquisition within pediatric cardiology medical education is developing [10, 27]. XR is already utilized readily to teach and promote practice of techniques in surgical specialties [28]. Recent changes to the pediatric residency requirements by the Accreditation Council for Graduate Medical Education have placed greater emphasis on more individualized components at the expense of certain procedural requirements during training [29, 30]. Pediatric cardiology remains a field in which procedural experience is critical. Incorporation of XR modalities into pediatric cardiology fellowship training programs may be necessary to provide learners with additional hands-on experience and improved sense of agency with procedures they may no longer receive as readily [31].

As the role of XR in pediatric cardiology medical education evolves, it will be critical to ensure new learning methods are validated for knowledge acquisition, retention, and dissemination. Pierick et al. have developed a validated assessment tool to assess learner comprehension following exposure to a hypoplastic left heart syndrome VR program for pediatric cardiology fellows [32]. Such tools are invaluable to ensuring that outcomes involving XR curricula are educationally meaningful and can be rigorously studied. Finally, the application of XR into pediatric cardiology medical education provides the potential for dissemination of educational material and experiences to under-resourced groups [33, 34]. With the advancements and future directions outlined, the role of XR in pediatric cardiology is promising and has the potential to revolutionize medical education.

## Procedural Planning

### Surgical Interventions

Accurate pre-operative understanding of cardiac anatomy is essential for optimizing outcomes in congenital cardiac surgery because intra-operative visibility is often limited. In complex cases, conventional 2D imaging may be insufficient for comprehensive anatomical understanding [1]. Multiple studies have demonstrated significant promise with XR technologies within this field. A single center study comparing various imaging modalities, including traditional 2D CTs, 3D printed heart models, and VR, found that 76% of cardiac specialists ranked VR as the most effective tool for presurgical planning and comprehension of spatial relationships [2]. Similarly, Gehrsitz et al. assessed CT and MRI based MR holograms in patients undergoing congenital cardiac surgeries versus matched controls without MR review. MR use significantly reduced intra-operative preparation time (mean of 58 versus 73 min, *p* < 0.05) [3].

XR is particularly valuable in surgical planning for complex CHD, wherein significant anatomical variations between patients often requires a customized approach. Double outlet right ventricle (DORV) is a prime example. Ye et al. found that MR planning in patients with DORV reduced preoperative planning time (mean of 52 versus 66 min, *p* < 0.05) and eliminated the need for intraoperative revisions, compared to 3 plan changes in the control group that relied solely on traditional 2D CT imaging [6]. Peek et al. found that in DORV patients, the feasibility of VSD patch closure was most accurately assessed using 3D-VR reconstructions (92%), compared to 3D-printed models (66%) and conventional ultrasound/CT (46%); this difference between modalities was statistically significant (*p* < 0.01) [35]. Another complex cardiac diagnosis with significant anatomic variability is pulmonary atresia with ventricular septal defect and major aortopulmonary collateral arteries (PA/VSD/MAPCAs). Qiu et al. divided patients with PA/VSD/MAPCAs into conventional imaging and 3D imaging groups before undergoing single-stage complete repair with unifocalization. In the 3D group, surgical planning was conducted with a CT-based VR model and 3D printed model; intraoperatively, an MR hologram was provided. The 3D group had shorter cardiopulmonary bypass times (mean 93 vs. 145 min, *p* = 0.099) and no early deaths, compared to 3 in the conventional group [7].

XR is also valuable for determining feasibility and cannulation strategies for mechanical circulatory support. It enhances understanding of the interactions between anatomy, myocardial and valvular motion, and flow dynamics. Ensuring proper device fit is essential for preserving atrioventricular valve function, minimizing structural distortion, and reducing the risk of low flow and pump thrombosis [36]. Davies et al. demonstrated the use of VR to optimize assessment of VAD pump positioning, inflow cannula angle, and the influence of chest wall anatomy in patients with single ventricle physiology and cardiomyopathy [37].

While 3D software typically relies on advanced imaging, XR can also integrate echocardiographic data. This is particularly useful for visualizing atrioventricular valve tissue and chordae, which are less well-defined on CT and MRI. Pushparajah et al. used echocardiographic data to generate 3D VR models of patients requiring atrioventricular valve surgery. In 67% of cases, surgeons reported increased confidence in understanding the pathology and surgical plan, with 53% indicating they would have made minor changes and 7% major changes to their approach based on VR visualization [38].

At our institution, we have incorporated virtual reality into our multidisciplinary case management conferences to facilitate pre-procedural planning for complex cases. Every case with advanced imaging is considered for presentation using the EchoPixel platform (EchoPixel Inc, San Jose, CA), which allows conference attendees to view interactive, holographic models of patient specific anatomy in real-time. A key feature of this platform is the use of “cut planes”, which allows conference members to virtually slice through cardiac structures and expose intracardiac relationships that may otherwise be difficult to visualize with traditional imaging. Our center has utilized this approach in a variety of challenging cases, including a patient with DORV, malposed great arteries, and hypoplastic left heart structures. The VR model clarified the approach to septation and baffle placement by enhancing understanding of the baffle pathway’s relationship to the outflows and atrioventricular valves. In another case involving an anomalous right coronary arising from the left sinus, a VR model provided clearer insight into the anomalous coronary course in relation to the valve commissure and surrounding structures, thus aiding in surgical decision-making. In addition, we have on occasion incorporated Elucis VR software (Realize Medical Inc., Ottwaw, ON, Canada) into our multidisciplinary case management conferences to allow surgeons to demonstrate their repair strategies in real-time on an interactive, virtual model. In one case, a surgeon used this platform to model common atrioventricular valve septation via a custom virtual patch placement. Another pre-operative plan was made for a patient with a hemi-Senning anatomy to guide the approach for connecting the SVC to the atrial baffle without undue tension. Beyond conference, surgeons continue to use Elucis to rehearse operations and mitigate modifiable risks. We have found that this immersive, interactive approach fosters improved understanding and supports precision in surgical planning.

## Transcatheter Interventions

XR applications are similarly impactful for catheter-based therapies. As the field of interventional cardiology continues to expand into the management of lesions that were once addressed surgically, VR has become an increasingly valuable tool. It promotes the development, validation, and refinement of emerging transcatheter techniques via a manipulable 3D understanding of patient-specific anatomy. Szeliga et al. applied VR to ductal stenting in neonates with ductal-dependent pulmonary circulation, improving assessment of ductal length, tortuosity, curvature index, and optimal access route, thereby enhancing procedural safety and operator confidence [39]. VR has also successfully been utilized for preprocedural planning in a newborn with heterotaxy syndrome, singly ventricle physiology, interrupted inferior vena cava, partial anomalous pulmonary venous drainage, and a restrictive atrial communication. VR was instrumental in planning jugular venous access for atrial septoplasty and contributed to procedural success [40]. Sadeghi et al. conducted a pilot study using CT-based VR to plan transcatheter paravalvular leak closure of moderate or greater aortic or mitral paravalvular leaks. Although device sizing most closely approximated CT-derived measurements, VR offered additional measurements, including the length of the lesion and proximal and distal orifice dimensions, aiding in device selection and procedural planning [41].

VR-based planning has had promising applications for transcatheter pulmonary valve implantation (TPVI). In blinded VR simulations performed by two interventional cardiologists on patients previously screened by commercial companies’ dedicated analyses for self-expanding TPVI evaluation, VR evaluation showed similarly acceptable TPVI candidacy of all patients accepted by manufacturer analysis using the Elucis VR software. Importantly, two patients who were not deemed candidates for TPVI by the companies were thought to be good candidates on VR screening and ultimately underwent successful TPVI, demonstrating potential additive utility in using VR technology to determine candidacy [42].

XR also holds promise for patient selection and procedural planning in patients with superior sinus venosus defects undergoing transcatheter covered stent correction. This cardiac lesion is particularly well-suited for XR visualization, as it requires comprehensive 3D understanding of the patient specific target anatomy and surrounding structures. Stephenson et al. evaluated patients previously identified as candidates for intervention based on conventional 2D CT imaging. Two blinded interventional cardiologists reviewed the pre-procedural CTs using Heart VR (a novel prototype VR system) to assess procedural feasibility and potential need for pulmonary vein protection. Three of 15 patients were found to be unsuitable candidates during the procedure. Comparatively, all cases that were deemed to be good candidates by VR (*n* = 12) proved to be technically feasible. VR correctly identified all cases requiring pulmonary vein protection (*n* = 5), though it overestimated this need in 7 of the 24 combined reviews by both interventionalists. Notably, both interventionalists reported increased confidence in case selection when using VR [43].

**Fig. 3:**
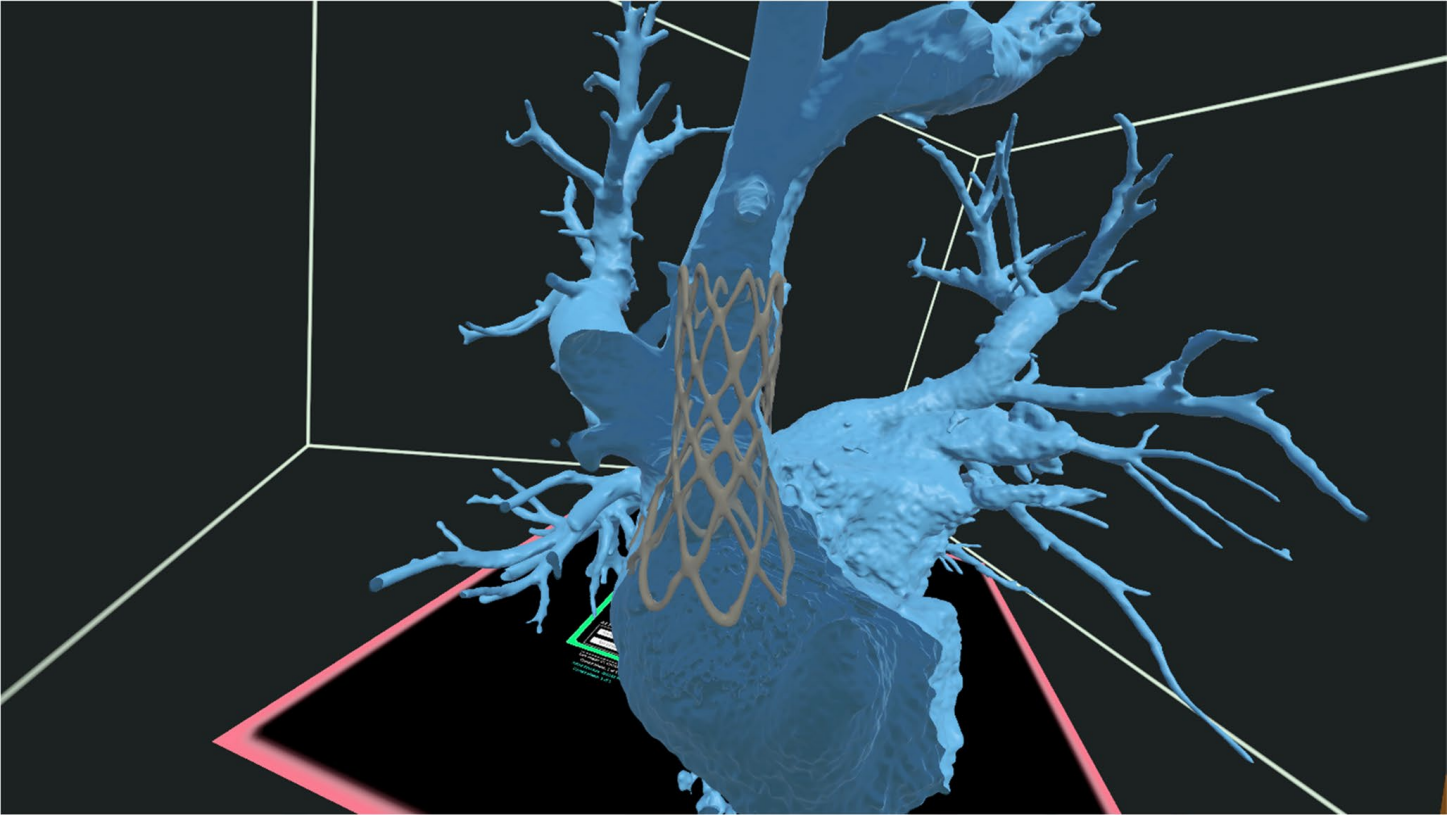
Virtual reality model of a superior sinus venosus defect created via Elucis, demonstrating covered stent placement to determine candidacy for transcatheter correction

Our institution has also integrated VR into the planning process of superior sinus venosus defect interventions. The Elucis platform has been used to perform virtual stent implantations to evaluate procedural candidacy and facilitate planning. Rotational videos of the model and stent placement were reviewed during multidisciplinary case management conferences to enhance discussion and decision-making (Fig. 3, Video 1).

Intraprocedural 3D visualization has been greatly advanced in the congenital catheterization laboratory by 3D Rotational Angiography (3DRA). The utility of 3DRA has been well-described, but it is limited in that the acquisition must be processed into a static 3D computer model and evaluated on a 2D computer screen. In 2016, Bruckheimer et al. demonstrated feasibility of using 3DRA and live 3D transesophageal echocardiography to create real-time interactive 3D digital holograms for intraprocedural use [44]. The feasibility of converting 3DRA acquisitions to AR models for visualization via wearable headsets during congenital cardiac catheterizations has also been demonstrated [45, 46]. In a study comparing AR visualization of 3DRA models to traditional computer models, there was majority agreement among investigators that AR models provided a superior appreciation of 3D relationships. AR was helpful in identifying pathology and assisting in planning in approximately 94% of cases in this study [46]. Szeliga et al. more recently demonstrated the integration of 3DRA into VR modeling to guide percutaneous and hybrid interventions in infants with pulmonary vein stenosis, which improved catheter maneuverability and balloon support, resulting in a shorter and more effective procedure. It also ultimately replaced the need for additional preoperative CT in one of the cases [47]. Together, these studies highlight the expanding role of XR in transcatheter interventions, offering enhanced anatomical insight and greater procedural confidence across a growing spectrum of CHD lesions.

## Navigating Challenges

Despite the growing promise of XR technologies, its integration into pediatric cardiology and cardiothoracic surgery comes with several challenges that must be addressed to ensure safe, effective implementation. Data privacy is a concern, particularly as XR platforms increasingly incorporate real patient data into immersive, interactive environments. Ensuring compliance with data protection regulations and maintaining secure data handling practices is essential [48, 49].

Financial barriers present another hurdle. Although XR technologies can be less expensive than the ongoing costs associated with 3D printed models, the cost of hardware, software, and technical support remains a limitation for many institutions. However, more affordable options, such as smartphone-based systems [50] are emerging and may improve accessibility [51].Validation gaps also persist. Although early proof-of-concept studies and case reports are promising, clinical effectiveness needs to be further investigated by large-scale, standardized outcome data [5, 51, 52].

Cybersickness, which is typically characterized by symptoms like headache, nausea, and dizziness, may limit prolonged use of these technologies, particularly in VR environments [52]. Similarly, technical issues such as image distortion or calibration errors can disrupt the user experience and reduce reliability [52].

Finally, like any new technology, XR comes with a learning curve. Initial reluctance among users, especially those less familiar with immersive platforms, may slow adaptation [53]. Ongoing training and intuitive user interfaces will be key to overcoming this barrier.

### Implementation of XR Technologies Within a Congenital Heart Center

XR technologies clearly fill important gaps within the field of CHD. In order to implement these technologies within a congenital heart center, one must first identify institutional workflow, knowledge, and clinical gaps that can be supplemented. XR “champions” are important to identify and often include one or more imaging experts, interventional cardiologists, CT surgeons, and Radiology Departments with common interests. Funding is needed for the early hardware costs (generally not recurring), and license costs for XR software, although free VR applications are available and institutions unknowingly may have access to VR software through other existing segmentation program licenses. Research and educational grants are valuable preliminary resources to starting an XR Program. The utility of XR applications should be shown early with valuable use cases of complex anatomy and procedural planning. When attempting to demonstrate utility prior to purchasing XR tools, collaborating with colleagues at other centers with XR Programs to help show these uses can be invaluable displays of impact.

## Future Directions

As XR continues to evolve, its role in CHD is expected to grow beyond pre-procedural planning and education. Moving forward, one major frontier is the integration of artificial intelligence (AI) with XR platforms. Currently, image segmentation, which is a foundational step in generating XR models, is often performed manually. This requires significant time and expertise and is subject to user variability and error. AI-driven segmentation, particularly utilizing deep learning algorithms, is a growing tool that can enable faster and more accurate model generation [54]. In addition to stream-lining segmentation, AI can enhance machine-human interaction within XR environments, potentially reducing cyber sickness and enabling gesture-based manipulation in sterile procedural environments [36].

Building on these advances, XR is now beginning to extend beyond preprocedural planning, with growing interest in its intraoperative applications. Mixed reality platforms now allow 3D models to be overlaid in the operative field, offering real-time anatomical guidance [55]. These tools have the potential to improve procedural accuracy, safety, and efficiency [51].

Looking ahead, insights from adult cardiology and other surgical fields can inform future XR applications in CHD. As the technology becomes more user-friendly and affordable, XR is increasingly positioned to become fully integrated into clinical workflows. It has the potential to transform every phase of care for patients with CHD, from planning and execution to training and education.

## Conclusions

XR is redefining the landscape of CHD by bridging key gaps in patient education, trainee learning, and procedural planning. Through immersive, patient-specific models, XR enhances understanding of complex cardiac anatomy and supports precise, individualized care strategies. Although larger, more comprehensive studies are needed to guide its future use, XR’s integration into clinical practice is accelerating and holds strong promise as a transformative tool across the continuum of CHD care.

## Key References

- Kieu V, Sumski C, Cohen S, Reinhardt E, Axelrod DM, Handler SS. The Use of Virtual Reality Learning on Transition Education in Adolescents with Congenital Heart Disease. Pediatr Cardiol. 2023;44(8):1856-60. doi: 10.1007/s00246-023-03292-w.
  - VR models improved patient understanding of their heart condition and surgery.
- Blonsky SE, Henning RE, Masterson EM, Axelrod DM, Handler SS, Yu S, Owens ST. Virtual Reality Curriculum Increases Pediatric Clerkship Students’ Knowledge of Congenital Heart Disease. Pediatr Cardiol. 2025. doi: 10.1007/s00246-025-03797-6.
  - Use of the Stanford Virtual Heart model with medical students improved performance on post-intervention assessments of CHD knowledge.
- Stephenson N, Rosenthal E, Jones M, Deng S, Wheeler G, Pushparajah K, et al. Virtual Reality for Preprocedure Planning of Covered Stent Correction of Superior Sinus Venosus Atrial Septal Defects. Circ Cardiovasc Interv. 2024;17(12):e013964. doi: 10.1161/CIRCINTERVENTIONS.123.013964.
  - In patients with sinus venosus atrial septal defects and anomalous pulmonary venous return undergoing transcatheter covered stent correction, VR models enhanced assessment of procedural feasibility and increased operator confidence.

## Supplementary Information

Below is the link to the electronic supplementary material.

Supplementary Material 1. Virtual reality evaluation of stent implantation to determine candidacy for transcatheter correction of a superior sinus venosus defect via Elucis software. (MP4 101 MB)

## Data Availability

Since this is a review paper, there is no data to make available.
